# COGNITIVE IMPAIRMENT IN VIETNAMESE OLDER ADULTS: EFFECTS OF LIVING ARRANGEMENTS AND FAMILIAL SUPPORT

**DOI:** 10.1093/geroni/igae098.0475

**Published:** 2024-12-31

**Authors:** Attrayee Bandyopadhyay, Kim Korinek, Daniel Adkins

**Affiliations:** University of Utah, Salt Lake City, Utah, United States; University of Utah, Salt Lake City, Utah, United States; University of Utah, Salt Lake City, Utah, United States

## Abstract

Living alone has emerged as a prominent modifiable risk factor for cognitive impairment and dementia. How living arrangements influence risk for cognitive disorder is less well understood in low-to-middle income countries (LMICs) where Alzheimer’s disease and related dementias (ADRD) burden is rising and falls heavily upon family members. Vietnam is one such LMIC where ADRD rates are rising as demographic and cultural changes alter the living arrangements pertinent to cognitive aging. In this paper, we analyze data from the Vietnam Health and Aging Study, a longitudinal study of 2,447 older adults aged 60 and above residing in four districts of northern Vietnam, to examine the association between living arrangements and cognitive impairment. In a series of multiple linear and ordered logistic regression analyses, we estimate cognitive function using an abridged version of the Mini-Mental Scale Examination and its association with a categorical measure of living arrangements, and mediated by measures of social support, stress, and socioeconomic status. Findings indicate that females generally have poorer cognitive function than males, particularly those living alone. By comparison, those living with both spouse and children exhibit higher cognitive functioning. Nested model comparisons suggest that the ill effects of living alone on cognitive function are due in part to weaker social support, greater stress, and poorer socioeconomic conditions as compared to those living with spouses and children. These findings suggest research on the etiology of cognitive impairment should address nuanced forms of family support and stress as they occur and diverge across living arrangement configurations.

